# Bouillonamide: A Mixed Polyketide–Peptide Cytotoxin from the Marine Cyanobacterium *Moorea bouillonii*

**DOI:** 10.3390/md11083015

**Published:** 2013-08-19

**Authors:** Lik Tong Tan, Tatsufumi Okino, William H. Gerwick

**Affiliations:** 1 Natural Sciences and Science Education, National Institute of Education, Nanyang Technological University, 1 Nanyang Walk, 637616, Singapore; 2 Division of Environmental Materials Science, Graduate School of Environmental Science, Hokkaido University, Sapporo 060-0810, Japan; E-Mail: okino@ees.hokudai.ac.jp; 3 Center for Marine Biotechnology and Biomedicine, Scripps Institution of Oceanography and Skaggs School of Pharmacy and Pharmaceutical Sciences, University of California, San Diego, La Jolla, CA 92093, USA

**Keywords:** marine cyanobacterium, *Mooreabouillonii*, polyketide–polypeptide, depsipeptide, cytotoxic

## Abstract

The tropical marine cyanobacterium, *Moorea bouillonii*, has gained recent attention as a rich source of bioactive natural products. Continued chemical investigation of this cyanobacterium, collected from New Britain, Papua New Guinea, yielded a novel cytotoxic cyclic depsipeptide, bouillonamide (**1**), along with previously reported molecules, ulongamide A and apratoxin A. Planar structure of bouillonamide was established by extensive 1D and 2D NMR experiments, including multi-edited HSQC, TOCSY, HBMC, and ROESY experiments. In addition to the presence of α-amino acid residues, compound **1** contained two unique polyketide-derived moieties, namely a 2-methyl-6-methylamino-hex-5-enoic acid (Mmaha) residue and a unit of 3-methyl-5-hydroxy-heptanoic acid (Mhha). Absolute stereochemistry of the α-amino acid units in bouillonamide was determined mainly by Marfey’s analysis. Compound **1** exhibited mild toxicity with IC_50_’s of 6.0 µM against the neuron 2a mouse neuroblastoma cells.

## 1. Introduction

Of the diverse secondary metabolites, the hybrid polyketide–polypeptide structural class constitutes a distinct and important feature of filamentous marine cyanobacterial secondary metabolism [1,2,3]. These molecules are characterized by a number of unique structural features, such as the incorporation of modified amino/hydroxy acids, heteroaromatic ring systems, as well as extended polyketide-derived units. In addition, many compounds have been reported to possess potent biological properties, ranging from antifungal (e.g., hectochlorin and lyngbyabellin B), cytotoxicity (e.g., largazole) to neurotoxicity (e.g., antillatoxin). Moreover, due to their specific interactions with cellular targets, such as microtubules and enzymes, these compounds are deemed attractive for further development as therapeutic drugs, particularly as anticancer agents. For instance, auristatin E (a synthetic derivative of dolastatin 10) has been formulated as an antibody drug conjugate, brentuximab vedotin, and approved recently for the treatment of Hodgkin lymphoma and anaplastic large cell lymphoma [4].

**Figure 1:**
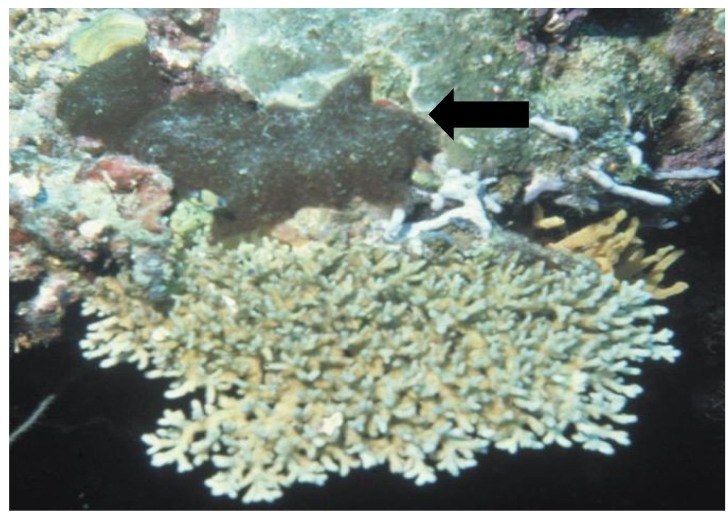
*Moorea bouillonii* found attached to corals.

Of the various strains of filamentous marine cyanobacteria, *Moorea bouillonii*, in particular, is gaining attention as a prolific producer of novel secondary metabolites. This cyanobacterium was first described in 1991 with type locality from Hansa Bay, Laing Island, Papua New Guinea [5]. Based on phylogenetic data, its taxonomy has recently been reclassified under the *Moorea* genus [6]. This cyanobacterial species occurs in shallow tropical waters and can be readily recognized by its thallus morphology, which can form tenacious mats found attached to either dead corals of the *Acropora* varieties or to other calcareous matter (Figure 1). To date, more than 20 compounds, with various biological activities, have been isolated from *M. bouillonii*. These molecules include macrolides, such as laingolide A, madangolide, and cyanolide A, and mixed polyketide-polypeptides, including apratoxins E–G, alotamide A, and lyngbyabellins K–N [7,8,9,10,11,12]. From an ecological perspective, these compounds could function as potential allelochemicals against competition from other benthic marine organisms [13].

**Figure 2:**
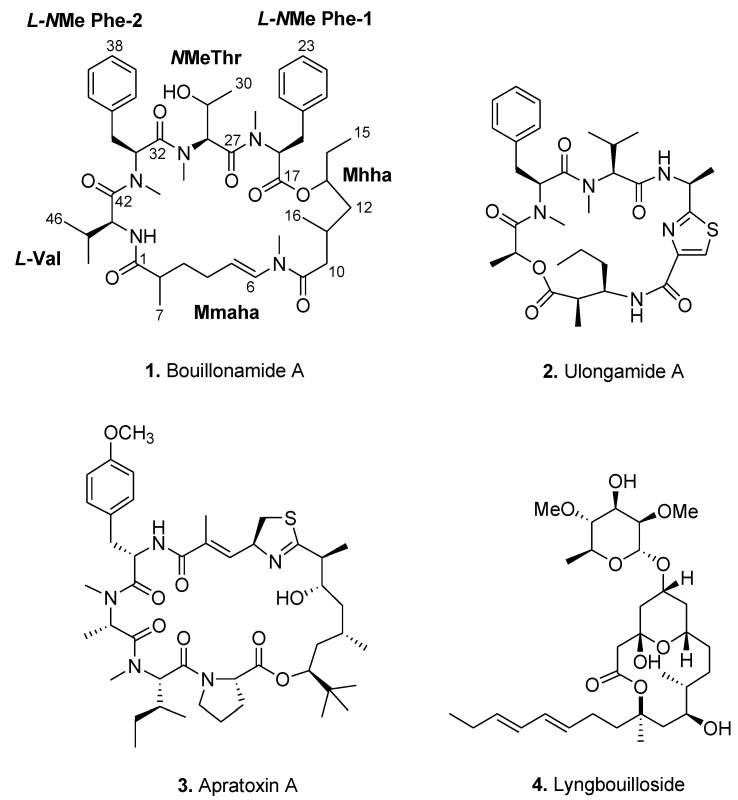
Natural products isolated from *Moorea bouillonii* (PNGRD 8/21/00-2).

In the course of our continued search for pharmaceutically-useful marine cyanobacterial compounds, we have identified the organic extract of *Moorea bouillonii* (PNGRD 21/Aug/00-2), collected from the northern coast of New Britain, Papua New Guinea, to be rich in secondary metabolites. Subsequent chemical investigation of bioactive fractions yielded a minor but novel cyclic depsipeptide, bouillonamide (**1**), along with the known compounds, ulongamide A (**2**) and apratoxin A (**3**) (Figure 2). In addition to these compounds, we have previously reported the isolation of a novel glycosidic macrolide, lyngbouilloside (**4**) (Figure 2), from the same organic extract of *M. bouillonii* (PNGRD 8/21/00-2) [14]. Herein, we describe the structural elucidation, including stereochemistry, of **1** and biological activities of compounds **1** to **3**.

## 2. Results and Discussion

High resolution FABMS of bouillonamide (**1**) (Figure 2) gave an [M + H]^+^ peak at *m/z* 818.5068 for a molecular formula of C_46_H_67_N_5_O_8_, requiring 16 degrees of unsaturation. The IR spectrum of **1** showed absorption bands at 1726 and 1637 cm^−1^ indicating the presence of both ester and amide functionalities, respectively. A strong absorption band was also observed at 3440 cm^−1^, suggesting the presence of an -OH group involved in intramolecular hydrogen bonding. A total of 46 carbon signals could be observed from the ^13^C NMR spectrum (Table 1) of **1** and these were defined as six carbonyls, fourteen olefinic carbons (assigned to two phenyl functional groups and one double-bond), nine sp^3^ methines (two bearing oxygen, δ 67.8 and δ 76.6), seven methylenes, and ten methyls. Four of the ten methyls belonged to *N*-methyl functional groups as revealed by H_3_ singlets at δ 2.79, δ 2.91, δ 3.08, and δ 3.10. Since a total of 15 degrees of unsaturation were accounted for from the 1D NMR data, bouillonamide was deduced to be monocyclic.

**Table 1:** NMR spectral data for bouillonamide (**1**) at 400 MHz (^1^H) and 100 MHz (^13^C) in CDCl_3_.

Unit	Position	δ_H_, mult. (*J* in Hz)	δ_C_	HMBC
Mmaha	1		175.7 s	
	2	2.34 m	42.3 d	C-1, C-7
	3	1.68 m	36.9 t	
		1.27 m		
	4	1.93 m	29.3 t	
		1.69 m		
	5	4.89 m	109.6 d	
	6	6.64 d (13.4)	130.5 d	C-4, C-5, C-8, C-9
	7	1.19 d (7.1)	20.5 q	C-1, C-2, C-3
	8 *N*Me	3.08 s	29.7 q	C-5, C-6, C-9
Mhha	9		171.3 s	
	10	2.44 m	39.5 t	C-9, C-11, C-12, C-16
		2.44 m		C-9, C-11, C-12, C-16
	11	2.03 m	28.0 d	
	12	1.64 m	40.5 t	C-10, C-1, C-13, C-14, C-15, C-16
		1.52 m		C-10, C-1, C-13, C-14, C-16
	13	4.96 m	76.6 d	C-15
	14	1.85 m	25.9 t	
		1.62 m		
	15	0.94 t (7.4)	9.2 q	C-13, C-14
	16	0.94 m	20.6 q	C-10, C-11, C-12
*N*Me-Phe-1	17		169.5 s	
	18	4.31 dd (11.5, 3.8)	61.4 d	C-17, C-19, C-26, C-27
	19	3.28 m	36.1 t	C-18, C-20, C-21/25
		2.90 m		C-18, C-20, C-21/25
	20		136.5 s	
	21/25	7.13 m	129.5 d	
	22/24	7.35 m	128.8 d	
	23	7.06 m	127.4 d	
	26 *N*Me	2.91 s	30.9 q	C-18, C-27
*N*Me-Thr	27		171.3 s	
	28	4.54 d (4.7)	57.7 d	C-29, C-30, C-31
	29	3.85 m	67.8 d	
	OH	3.34 d (4.7)		C-29, C-30
	30	0.84 d (6.2)	19.9 q	C-28, C-29
	31 *N*Me	2.79 s	32.8 q	C-32, C-28
*N*Me-Phe-2	32		170.4 s	
	33	5.49 dd (8.4, 5.6)	56.4 d	C-32, C-34, C-41, C-42
	34	3.28 m	35.3 t	C-32, C-33, C-35, C-36/40, C-42
		2.90 m		C-32, C-33, C-35, C-36/40, C-42
	35		138.0 s	
	36/40	7.23 m	129 5 d	
	37/39	7.35 m	129.3 d	
	38	7.24 m	127.7 d	
	41 *N*Me	3.10 s	31.3 q	C-33, C-42
Val	42		172.1 s	
	43	4.86 dd (8.6, 3.0)	52.8 d	C-42, C-44, C-45, C-46
	44	1.96 m	31.2 d	
	45	0.74 d (6.7)	16.4 q	C-43, C-44, C-46
	46	0.95 d (6.8)	20.6 q	C-43, C-44, C-45
	*N*H	6.02 d (8.6)		C-42, C-1

**Figure 3:**
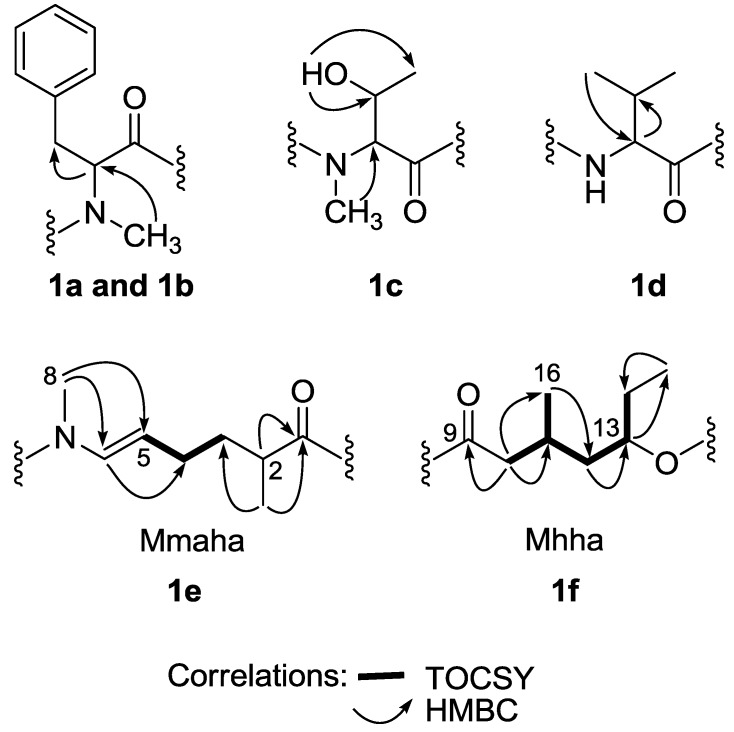
Partial structures **1a** to **1f** of bouillonamide (**1**).

Six partial structures were generated using a combination of multi-edited HSQC, HMBC, and TOCSY experiments (**la** to **1f** in Figure 3). These included two *N*-MePhe residues, one *N*-MeThr residue, one Val residue, a 2-methyl-6-methylamino-hex-5-enoic acid (Mmaha) moiety, and a 3-methyl-5-hydroxy-heptanoic acid (Mhha) unit. The presence of an enamide functionality in bouillonamide (**1**) was deduced from HMBC correlations from the *N*CH_3_ proton signal (H-8, δ 3.08 s) to the carbonyl carbon at C-9 (δ 171.3) and to the vinyl carbons at C-5 (δ 109.6) and C-6 (δ 130.5). Further HMBC and TOCSY data indicated that the enamide was part of a 2-methyl-6-methylamino-hex-5-enoic acid (Mmaha) moiety. These included HMBC correlations from the olefinic proton H-6 (δ 6.64 d) to C-4 (δ 29.3); the methyl protons at H_3_-7 (δ 1.19 d) to C-1 (δ 175.7), C-2 (δ 42.3), and C-3 (δ 36.9) as well as TOCSY correlations between the olefinic proton at H-5 (δ 4.89 m) and the methylene protons at H_2_-4 (δ 1.93 m, δ 1.69 m); and between the methylene protons at H_2_-3 (δ 1.68 m, δ 1.27 m) and H_2_-4 (δ 1.93 m, δ 1.69 m) (Figure 3). The configuration of the C-5-C-6 double bond was assigned as *E* on the basis of a 13.4 Hz coupling constant. 

A 3-methyl-5-hydroxy-heptanoic acid (Mhha) unit in **1** was deduced from HMBC data (Figure 3). Key HMBC correlations included H_2_-10 (δ 2.44 m) to C-9 (δ 171.3), C-11 (δ 28.0), C-16 (δ 20.6), and C-12 (δ 40.5); H_3_-16 (δ 0.94 m) to C-10 (δ 39.5), C-11 (δ 28.0), and C-12 (δ 40.5); H_2_-12 (δ 1.64 m) to C-13 (δ 76.6) and C-14 (δ 25.9); H-13 (δ4.96 m) to C-15 (δ 9.2); and H_3_-15 (δ 0.94 t) to C-14 (δ 25.9). In addition, the ^1^H-^1^H spin systems from H-10 to H-14 could be observed in the TOCSY data (Figure 3). Connectivity of these six partial structures was achieved from HMBC and ROESY data. Useful correlations from either the *N*H or *N*CH_3_ proton signals to carbonyls of adjacent partial structures were observed from HMBC data, which included correlations from *N*CH_3_-26 to C-27, *N*CH_3_-31 to C-32, *N*CH_3_-41 to C-42, Val-*N*H (δ 6.02) to C-1, and *N*CH_3_-8 to C-9, establishing the sequence of *N*-MePhe-1/*N*-MeThr/*N*-MePhe-2/Val/Mmaha/Mhha. Although there was no HMBC correlation linking the Mhha unit and *N*-MePhe-1 residue, a ROESY correlation between H_3_-15 of the Mhha unit and the β-protons (H_2_-19) of the *N*-MePhe-1 residue was observed, thereby closing the ring structure as shown in **1**.

The absolute configuration of the *N*-MePhe and Val residues in bouillonamide (**1**) were determined by Marfey’s method and showed the presence of l-forms of these amino acids [15]. The relative stereochemistry of the *N*-MeThr unit in **1** was established using ROESY data which showed strong correlations between the αH (H-28) of *N*-MeThr and the -OH as well as the αH (H-18) of *N*-MePhe-l, indicating that they lie on the same side in the molecule and hence having the relative configuration of 28S*. Shortage of material prevented stereochemical determination of the Mmaha (C-2) and the Mhha (C-11 and C-13) units in **1**.

The biosynthesis of bouillonamide (**1**) appears to involve a hybrid of polyketide synthase and polypeptide synthetase pathways. The presence of two unusual units of Mmaha and Mhha in **1** poses an intriguing question concerning their biosynthetic origins. These units could be initiated by the incorporation of a propionyl unit and after two acetate extensions, a glycine and two additional acetate units are condensed into the linear chain with methionine incorporation [16]. It is interesting to note that the enamide functionality in bouillonamide (**1**) is present in two other *L. bouillonii* compounds, laingolide and laingolide A, as well as in the scytophycins, a class of potent cytotoxic cyanobacterial metabolites [7,17,18]. 

Bouillonamide (**1**) and ulongamide A (**2**) were evaluated for biological activities in the brine shrimp (*Artemia salina*) toxicity and the neuro-2a mouse neuroblastoma cells cytotoxicity assays. In the former assay, **1** and **2** exhibited LD_50_s’ of 9.0 µM and 18.0 µM, respectively, while IC_50_s’ of 6.0 µM and 16.0 µM, respectively, were observed in the latter assay. The known compound, apratoxin A (**3**), was also found to be cytotoxic with an IC_50_ of 1.0 µM when tested against neuro-2a mouse neuroblastoma cells.

## 3. Experimental Section

### 3.1. General Experimental Procedures

NMR experiments were measured on a Bruker AM 400 MHz NMR spectrometer in CDC1_3_ as an internal standard. Chemical shifts are reported in ppm and coupling constants (*J*) are reported in Hz. High resolution mass spectra were recorded on a Kratos MS50TC mass spectrometer. Optical rotation was measured on a Perkin-Elmer 141 polarimeter. UV and IR spectra were recorded on Beckman DU^®^ 640B and Nicolet 510 spectrophotometers, respectively. The isolation of compounds **1** to **4** was performed on a Waters Millipore^®^ Model 590 Pump and detected with a Waters Millipore^®^ Lambda-Max Model 480 LC spectrophotometer. All Marfey-derivatized products were analyzed using dual Waters 515 HPLC Pump and Waters 996 Photodiode Array Detector. 

### 3.2. Biological Material

Samples of the marine cyanobacterium, *Moorea bouillonii* Hoffman and Demoulin, were hand-collected at 8–15 m water depth, using SCUBA, from various reef systems at the northern coast of New Britain, Papua New Guinea (August 21–27, 2000). These localities were: Bangkok Pass (S 4°15.758′, E 151°28.547′), Father’s Reef (S 4°55.153′, E 150°54.554′), May Reef (S 5°13.692′, E 150°30.078′), Unea Island (S 4°50.783′, E 149°09.174′) and Long Island (S 5°14.528′, E 147°02.058′). Upon collection, the pooled marine cyanobacterial samples were preserved in 50% isopropyl alcohol and seawater and stored at low temperature until work-up. Voucher specimens are available from WHG as collection number PNGRD 8/21/00-2.

### 3.3. Extraction and Isolation of Bouillonamide **(1)**

The thawed cyanobacterial material was homogenized in CH_2_Cl_2_/MeOH (2:1, v/v), filtered, and the solvents removed *in vacuo* to yield a residue which was partitioned between CH_2_Cl_2_ and H_2_O. The marc was extracted repeatedly (4×) with CH_2_Cl_2_/MeOH (2:1, v/v) and the combined CH_2_Cl_2_ layers reduced *in vacuo* to yield about 2.0 g of a dark green extract. The crude extract was fractionated using normal phase silica gel (TLC grade) vacuum liquid chromatography (VLC) through a step-wise gradient solvent system of increasing polarity starting from EtOAc in hexanes to EtOAc in MeOH. Fractions eluting with 2% MeOH in EtOAc were found to be active at 10 ppm in the brine shrimp toxicity assay. This material was refractionated using Mega Bond RP-18 Sep Pak. The most active fraction (82% cytotoxicity at 10 µg/mL against the neuro-2a neuroblastoma cell line) was eluted with 20% H_2_O in MeOH, and then further purified by HPLC [Phenomenex LUNA 5µ Phenyl-hexyl 250 × 4.60 mm, CH_3_CN/H_2_O (61:39); detection at 220 nm] to give a slightly impure metabolite **1** and apratoxin A (**3**, 2.2 mg) [19]. Two more HPLC purification steps [(a) Phenomenex SYNERGI 4µ MAX-RP 80 A 250 × 4.60 mm, MeOH/H_2_O (83:17); (b) Phenomenex SPHERECLONE 5µ ODS (2) 250 × 10.00 mm, MeOH/H_2_O (90:10); detection at 220 nm] were necessary to give a pure preparation of bouillonamide (**1**, 1.0 mg). A second active fraction obtained from Mega Bond RP-18 Sep Pak was subjected to further HPLC purification on Phenontenex Sphereclone 5µ ODS (2); MeOH/H_2_O (82:18) to yield ulongamide A (**2**, 3.5 mg) and the glycosidic macrolide, lyngbouilloside (**4**, 4.5 mg) [14,20].

Bouillonamide (**1**): white amorphous solid; [α]^25^_D_ −178° (*c* = 0.19, CHCl_3_); UV (MeOH) λ_max_ 240 nm (ε 12400); IR (KBr) 3440, 2964, 2933, 1726, 1637, 1457, 1403, 1083, 1044 cm^−1^; LR FABMS *m/z* 818 (93), 800 (21), 774 (10), 140 (42), 134 (100); HR FABMS (positive ion, 3-nitrobenzyl alcohol) *m/z* obs. [M + H]^+^ 818.5067 (C_46_H_68_N_5_O_8_, −0.3 mmu dev.); ^1^H NMR (400 MHz, CDC1_3_) and ^13^C NMR (100 MHz, CDC1_3_) see Table 1.

### 3.4. Acid Hydrolysis and Marfey’s Analysis of Bouillonamide **(1)**

Bouillonamide (**1**, 200 µg) was placed in a pressure tube with 1 mL of 6 N HCl and hydrolyzed in a microwave oven (setting at high) for 1 min. Upon cooling and concentrating to dryness under a stream of N_2_ gas, the hydrolysate was dissolved in 50 µL of H_2_O followed by a 1% (w/v) solution of 1-fluoro-2,4-dinitrophenyl-5-l-alanine amide (Marfey’s reagent) in acetone (100 µL) and 20 µL of 1 M NaHCO_3_. After heating the mixture at 38 °C for 1 h, the reaction was cooled and acidified with 20 µL of 2 N HC1, and evaporated to dryness under N_2_ gas. The resulting products were redissolved in DMSO/H_2_O (1:1, 100 µL), and aliquots were subjected to reversed-phase HPLC analysis (Waters Nova-Pak^®^ C_18_, 3.9 × 150 mm) using two different solvent systems: (**A**) CH_3_CN–0.05% TFA in H_2_O linear gradient (10%–50% over 60 min) and (**B**) CH_3_CN–50 mM NH_4_OAc linear gradient (10%–50% over 60 min). The retention time (*t*_R_) of the derivatized amino acids in the hydrolysate of **1** matched those of l-Val (29.1 min; d-Val, 35.2 min in solvent system **A**) and l-*N*-MePhe (34.2 min; d-*N*-MePhe, 34.9 min in solvent system **B**).

### 3.5. Brine Shrimp Toxicity and Cytotoxicity Assays

Biological evaluations of chromatographic fractions and the pure compounds **1** to **3** for brine shrimp (*Artemia salina*) and neuro-2a neuroblastoma cell toxicity were performed as detailed previously [14].

## 4. Conclusions

A novel cyclodepsipeptide, bouillonamide (**1**), along with known compounds, ulongamide A (**2**) and apratoxin A (**3**), were isolated from the marine cyanobacterium, *Moorea bouillonii*, collected from Papua New Guinea. When tested against neuro-2a mouse neuroblastoma cell line, bouillonamide showed moderate cytotoxic activity with an IC_50_ of 6.0 µM, while apratoxin A exhibited significant activity with an IC_50_ of 1.0 µM. The isolation of these compounds further attests to the chemical richness of *M. bouillonii* as a source of novel bioactive molecules in drug discovery efforts.
